# Military Wives' Transition and Coping: Deployment and the Return Home

**DOI:** 10.5402/2012/798342

**Published:** 2012-07-14

**Authors:** Suzanne Marnocha

**Affiliations:** College of Nursing, University of Wisconsin Oshkosh, Oshkosh, WI 54901, USA

## Abstract

The objective of this qualitative study is to explore the experiences of wives of deployed soldiers. Semistructured interviews were used to answer the research questions. Meleis' Transitions Theory was used to guide the understanding of the wives' experiences. Phase One: news of deployment, property of awareness, themes of emotional chaos and making preparations. Phase Two: during deployment, property of engagement, themes of taking the reins and placing focus elsewhere, along with the property of change and difference, with themes of emotional and physical turmoil, staying strong, and reaching out. Phase Three: after deployment, property of time span, themes of absence makes the heart grow fonder and reestablishing roles. The study concluded that the wife often feels forgotten during deployment. Nurses can give better care by understanding how the different phases of deployment and separation affect the wife's coping ability and her physical and emotional health.

## 1. Introduction

The family experiences significant stress when a loved one is deployed to combat zones and exposed to dangerous operations. Approximately 1.5 million American troops have been deployed; one-third of these troops served at least two tours in a combat zone, 70,000 were deployed three times, and 20,000 were deployed at least five times. More than 3,240 American troops have died, and over 23,000 have returned home with physical wounds, psychological injuries, and permanent disabilities [1].

There is an abundance of information in the literature describing the human experience of the soldier's deployment, but little information or attention has been given to the deployment's effect on the wife. Fifty percent of military personnel are married, and nine out of ten military spouses are female [2]. Dealing with deployment can be an overwhelming process for the wife, as she deals with loneliness, loss of emotional support, pressure of an extensive separation, shifting responsibilities, potential difficulty in dealing with the children, financial constraints, and constant disruption of family roles [1, 3]. In addition to these stressors, her husband is being deployed to a combat zone where his life is threatened. The literature reflects that both wives and husbands of deployed soldiers exhibit different stages of grief and loss, including denial, anger, bargaining, depression, and acceptance [3–5]. Other significant emotional problems, such as anxiety [6, 7] and relationship dysfunction [1, 8], along with related health problems may occur. Returning home is also a particularly stressful time for the wife, since she must deal with reintegrating her husband into the family. Throughout the various phases of deployment, it is imperative that the wife learns strategies to reduce or minimize stressors and maintain her health and well-being [9, 10].

Wives of soldiers before, during, and after deployment are in a state of transition and vulnerability. The Transitions Theory [11] was chosen to help understand the experiences of these women. The purpose of this qualitative study was to explore community-dwelling military wives' perceptions of transition, adaptation, and coping with deployment.

## 2. Methods

The University of Wisconsin Oshkosh Institutional Review Board (IRB) for the Protection of Human Participants granted approval to conduct this study. The researcher performed in-depth interviews with 11 wives of deployed Army Reserve military members. Women were obtained by snowball sampling; one woman was approached by the researcher and she recommended others. The sample included White women, aged 22 to 42 years, living in the Midwest, whose husbands had been deployed one or more times during recent military conflicts. The women were highly educated; three had high school diplomas, two had completed 3 years of college, four had completed baccalaureate degrees, and two had masters degrees. Seven of the women had at least one child at home. Despite five of the wives being full stay-at-home moms before deployment, all began working either full- or part-time jobs outside of the home in addition to taking over duties of single parenthood. Six of the soldiers had been on one deployment and five of the soldiers had been on multiple deployments during the most recent conflicts in Iraq and Afghanistan. Ten of the soldiers had experienced direct conflict and one served in a healthcare role providing training and care for wounded soldiers. All wives spoke of their husbands returning intact physically, but also described lasting emotional issues. Sampling continued until data saturation was reached.

A researcher-developed interview guide consisting of eight open-ended questions was used for data collection to ensure consistency among participants. Each wife was asked to describe her marriage, along with the experience of deployment and her feelings corresponding to the deployment. Additional standardized probing questions were asked to more clearly define the experience, leading to detailed accounts of the experience of deployment and reintegration for each family. The researcher noted and systematically recorded nonverbal expressions during the interviews. Each interview was conducted in the participant's home or a quiet office setting, with each lasting approximately 60 minutes.

## 3. Data Analysis

Transitions Theory [11] was used to guide the overall understanding of the results. The properties of the theory consist of awareness, engagement, change and difference, time span, and critical points and events. These properties will be used to describe the study results [11, 12].

The data analysis followed an adaptation of the seven-step method for qualitative analysis developed by Collazzi [13]. The author first taped-recorded and then transcribed the narrative and field notes after each interview in order to capture the ambient environmental and nonverbal emotional tone. The author then listened to each of the interview tapes twice while reading the transcribed narrative and highlighting significant statements. Each of the highlighted statements was analyzed to determine explicate meanings. The predominant themes in each interview were then examined, which resulted in common themes appearing throughout all interviews. To establish rigor in qualitative research, the researcher undertook member-checking with three participants. Member-checking is used to confirm the dependability of qualitative data through discussions with a sampling of participants [14]. After reviewing the study results, three participants overwhelmingly agreed with the analysis and conclusions.

## 4. Results

Three phases of deployment, each with important themes, were recognized: (a) news of deployment, (b) during deployment, and (c) after deployment (Table 1).

### 4.1. Phase One: News of Deployment

#### 4.1.1. Property: Awareness

The property of awareness is related to perception, knowledge, and recognition of a transition experience. The level of awareness is often reflected in the degree of congruency between what is known about the process and responses and what constitutes an expected set of responses and perceptions of individuals undergoing similar transitions. News of deployment was defined as the timeframe from notification to departure. Service members were often busy training for the upcoming mission and preparing equipment for deployment [1]. Emotional chaos and making preparations were the two themes identified during this phase.

#### 4.1.2. Theme: Emotional Chaos

One of the themes emerging under Phase One: news of deployment was emotional chaos, which included not only anxiety, but also a wide range of other emotions. Separation and loss are anticipated by the entire family. A newly married wife remarked, “It happened really fast. We had a phone right next to the bed and when I picked it up it said US government. Oh boy, here we go, I could not believe it.” Another husband was deployed 1 month after their baby was born, leaving the wife to care for the infant alone while she was finishing school. “I couldn't believe it. We were just getting settled as a family and I was trying to go back (to school).” In general, most participants felt that the increased stress of daily life, with the additional responsibilities and the loss of a partner's help, was overwhelming. Several participants cried during this part of the interview.

#### 4.1.3. Theme: Making Preparations

The wives' facial expressions would change when relating this next theme. Many would have frowns of concentration or simply become more focused as they related making plans and the preparing for their husbands to leave. Several women reported additional requirements for weekend or two-week special training sessions which required their husbands to leave the family suddenly:

*We had been planning a party for (child's name) and he (the husband) could not come. It was so sad. I knew he needed to go (to training) to be ready but it was so sad. I know he felt bad too.*

Several women spoke of finishing up tasks around the house.

*When he found out he was going to be deployed he wanted to finish up a lot of projects. He decided to put in a new hot water heater. He knew we would be more comfortable knowing those types of things were taken care of.*

The wives related that there was the continuing pressure of maintaining outside work and concerns about money. “He kept working the night shift as a police officer and even tried to pick up extra shifts for money. He was always thinking about us.” The participants reported thinking about how they might handle the children on their own. A young woman with several small children spoke about “calling her mother to arrange for possible child care.” Participants reported that the military directed them to have discussions about spiritual issues and preparing wills. One young wife spoke of the military resources given to them. “The military guidelines said we had to discuss dying and who would care for the children. We had never done that before. It seemed odd, being such a young couple.”

Emotional chaos and making preparations were reported by most of the wives in this study as difficult for the entire family. As their husbands were preparing for the deployment, all women reported their workload increased tremendously.

### 4.2. Phase Two: Deployment

Deployment was described as the period from departure to return. During the deployment phase, two properties and five themes emerged. The property of engagement included the themes taking the reins and placing focus elsewhere. The property, change, and difference included the themes of emotional and physical turmoil, staying strong, and reaching out. 

#### 4.2.1. Property: Engagement

Engagement indicates the degree of personal involvement in the process.

#### 4.2.2. Theme: Taking the Reins

In taking the reins, the wife took over primary responsibility for herself and the family. Duties that were the husband's responsibility, such as bill paying, car repair, and home maintenance, became her responsibilities. This caused feelings of being overwhelmed, aggravated, and disorganized. A reoccurring theme which emerged was being the sole responsible adult for the children with little relief. One participant noted,

*Taking the reins was very much a challenge … to get through the stress of the day … (there was no one) to relieve, to give me a half an hour to just breathe without children and to have time alone. I had a lot more responsibility and had to take over.*

One wife explained that she coped with the deployment and taking over her husband's responsibilities through her strict routine. She said,

*I worked all the time … and that was my outlet … if I had one piece of advice to any woman or any person that has a loved one deployed, exercise, exercise, exercise, because the endorphins help your emotions, and to get confidence … I really balanced work and exercise with my social life, family, and friends as best I could and I think I did a great job so that got me emotionally pretty solid.*

#### 4.2.3. Theme: Placing Focus Elsewhere

During the phase of placing focus elsewhere some wives coped with deployment by practicing avoidance. Listening to news about the war was difficult, since they felt vulnerable and knew if a death or injury occurred it could have been their husband. Ignoring media reports was a coping mechanism for some of the wives. Several wives indicated watching the television or listening to the radio reports of military actions tended to create increased stress and sadness. One wife summed up what was evident in many of the women's commentary, “He got hit three times by roadside bombs. I only knew about one because I told him if he doesn't have to tell me, I don't want to know.” One mother with a middle school child said, “Whenever the news came on, I turned it off.” Other wives coped by focusing on their children or by socializing and getting out of the house.

*I always made sure I had plans on the weekends with friends or family. I think that was very important for my personal well-being and mental health or just to not go nuts with the children just being in that place by myself.*

One young woman who had no children stated, “I became a social butterfly. I went out all the time. I had a blast. I went on vacations.”

#### 4.2.4. Property: Change and Difference

Change may be related to critical or disequilibrating events, or to disruptions in relationships, routines or ideas, perceptions, and identities. Confronting difference is exemplified by unmet or diverging expectations, feeling different, being perceived as different, or seeing the world in different ways.

#### 4.2.5. Theme: Emotional and Physical Turmoil

Women reported a range of emotional and physical turmoil. One woman whose husband had been on multiple deployments stated,

*You're so full of emotions and even sometimes I think the best word is “numb.” I thought, what am I going to do? I could not do anything and felt stuck. You just have to go with it and I felt like this would break me down. I felt sick all of the time.*

A young working mother reported, “I developed terrible headaches and finally went to the doctor. No one asked me about my husband or deployment. I did not tell them. They gave me pills and told me to come back. I did not go back.”

Other indicators of emotional turmoil related to how the wife would manage total care of the home and children and maintain an outside job. One wife stated, “I was completely overwhelmed. The first thing I did is quit my part-time job. I just instantly called them and said “I'm done,” because there's nobody to … care for my children.” Several wives felt that the income reduction changed their lives, while others reported that active duty pay was welcomed, financially.

In the midst of their own distress, military wives were often worried and uncertain about how to respond to their children's emotional needs. One wife said, “The hardest thing was trying to explain to my 2-year-old where daddy was.” Another wife explained,

*I tried to explain where daddy had gone to my 4-year-old son, he just doesn't understand. I don't want him to be angry with daddy. I mean, it's his job and it's something that together he and I have chosen to do.*

Another mother indicated that she “… protected them (her children) from news stories about war.”

#### 4.2.6. Theme: Staying Strong

In all phases of deployment, staying strong played a major role.“You're just stuck and you have to go with it. I could either let this break me down or I can let it make me stronger, and that's just what I did.” Another wife (an Army reserve solider herself), whose husband had been deployed previously stated, “I thought I must be strong for him … He's still going to have to leave regardless of how much I cried or how mad I was or how pissed off I was with the Army.” Several wives found the midpoint of deployment as the time of greatest stress. Loneliness was an issue during deployment.

*He had been gone about 6 months when I had this horrible feeling come over me at night. I just could not believe how extremely lonely I felt. It was like a wave crashing over me. I decided I had to buck up and stay strong in order to survive.*

As wives learned to respond and adjust to new challenges, they developed new routines, a sense of independence, and improved self-confidence. Wives learned to effectively manage emotions and use their support systems to help them stay strong.

#### 4.2.7. Theme: Reaching Out

Since reaching out was a very important theme for women during deployment, all forms of communication became vital to the wife and family. Most military couples are in contact weekly by telephone or email, but women voiced that email and phone calls were “not enough.” “I tried to stay emotionally attached to him while he was over there, but it's hard to do that when you only get to talk 10 minutes, 2 to 3 times per week. Email … there is no emotion in email.” One woman spoke of finding strength and peace from her spiritual practice. “I did more praying before, during, and after the deployment than I've ever done in my 28 years of life. I had just one prayer … to keep him safe and bring him home in one piece.” Women spoke of the importance of the military support systems they used:

*We had a family readiness group—his battalion has a website and I was on it all the time. They allowed us to stay in touch though email. Sometimes he would write everyday and then just disappear for days or weeks.*

### 4.3. Phase Three: After Deployment

#### 4.3.1. Property: Time Span

The property of time span is characterized by flow and movement over time. There is an identifiable point, a period of instability, confusion, and distress, to an eventful ending with a new beginning or a period of stability. Some transitions are associated with an identifiable marker event, whereas others are not as evident. In some studies, critical points were often associated with increasing awareness of change or differences or more active engagement in dealing with the transition experiences.

When the husband arrived home, the major themes of the research became absence makes the heart grow fonder, with the property of time span, and reestablishing roles, with the property of critical points and events. Women reported that the emotions associated with deployment kept returning even after he came home and that reestablishing family roles was very difficult for both the wife and husband.

#### 4.3.2. Theme: Absence Makes the Heart Grow Fonder

Participants discussed the fact that absence actually made the relationship stronger and increased their desire for intimate time with their husbands, along with spending special time together with their children. One woman, who had previously experienced deployments, said,

*Overall, deployment was both positive and negative. Negative because it broke our family in a way … I'd say we're probably closer now than we were. I think we share a deeper communication now. We have both shared more than before he left.*

Another woman, also with previous deployment experience, stated,

*I think we are more grateful for our time together. He's home and we're spending more time together instead of a lot of times when we used to get caught up with our own lives. He wants to do things together as a family and with the kids. Before, he spent more time at work.*

Research demonstrates that husbands are much more sensitive and considerate during the reunion phase [2]. A young mother stated,

*Now when I face leaving him to go to work, I always stop and think how I felt when he was gone and the emotions come back to me again …. I guess you get sensitized to that certain event and you will always remember how you felt.*

A newly married woman stated, “I think it really makes you grateful for what you had that you don't realize it until it's gone.” 

#### 4.3.3. Property: Critical Points and Events

Women reported a period of uncertainty in which there were a number of critical points. Each of these critical points requires the nurse's attention, knowledge, and experience in different ways. During these periods, there is heightened vulnerability during which the wives encountered difficulties with self-care and care giving. The property of critical points and events is often associated with increasing awareness of change of difference or more active engagement in dealing with the transition experience. The final critical points are characterized by a sense of stabilization in new routines, skills, lifestyles, and self-care activities.

#### 4.3.4. Theme: Reestablishing Roles

Women verbalized a variety of emotions and issues surrounding reestablishing roles, ranging from, “getting used to another person in the home” and “making room for him in our home,” to changes in financial and child rearing responsibilities. Some participants noticed that they argued or nit-picked at each other about how things were done. “We started arguing about stupid things like how to load the dishwasher. I mean, ee-gads, the dishwasher. It sometimes escalated into something big.”

*I think the biggest arguments we have now are about finances. I did it all the time (managed money) when he was gone and now he has his own debit card and it's hard to figure out the checking account when he doesn't tell you what he spends.*

The most emotionally laden discussions came when the participants found that their children were resistant to following their dad's direction. A mother described her experience, “He is an officer so he just says, “Do this” and it gets done. He doesn't understand that a 2-year-old doesn't follow orders like those guys do.” Another young mother spoke while crying,

*He would scold (the toddler) for something that I would have scolded her for, but my mother-bear instincts kind of started to erupt because I was the only one that had done it for 14 months. So I had to be really careful of how I reacted to that—what my facial expressions were and everything. So I would just take a deep breath and say, “Daddy's right,” you know, and she'd be like “Oh.” And it was hard for her. She would immediately look at me, like “Can he talk to me like that? Can he tell me, no I can't do that?”*

After this young woman shared this information, she began crying and left the room for a few minutes to compose herself. I inquired whether she feels safe in her relationship and she said, “Yes, but we are in counseling.”

## 5. Discussion

The Transitions Theory [11] provided the framework to understand the complex narratives of these wives. The first phase was news of deployment and yielded two themes, emotional chaos and making preparations. During the emotional chaos phase, the wife experiences feelings of numbness, shock, denial, and disbelief, and may continue into protest and anger about the deployment experience [2, 4]. Wives may experience a wide range of emotions, including apprehensiveness, irritability, tenseness, resentment, emotional detachment, and marital disagreements [15, 16].

Literature supports that the second theme, making preparations, is an important phase. Both spouses are preparing for deployment in their own ways. According to the military, planning for how the wife will carry on during the deployment should occur before the separation happens [17]. According to the literature, a well-prepared family has the ability to respond with resilience when faced with recurring stressors, daily irritations, or traumatic situations. The couple is most adaptable during deployment when they are flexible, have good coping skills, a positive attitude, and receive community and social support [1]. Some common dynamics that can put military families at risk include rigid coping skills; family difficulties; young families, especially those experiencing a first military separation or those with young children and those with lower pay grades [1].

Advanced preparation, along with honest, open communication, is important for assisting the family with deployment [1]. Assisting the military wife in developing skills to handle this stressful time, along with the knowledge needed to refer them to other critical support resources, is part of the preparation. The Personal & Family Readiness Checklist [15] has been prepared to assist families facing deployment. Family readiness and support are priorities for every branch of the Armed Forces. The Office of Reserve Affairs [15], for example, has a 63-page booklet discussing how the individual and family should prepare for deployment, including finances, legal affairs, estate planning, family support resources, departing and returning to a career, relocation resources, and web links. The couple should also discuss who to contact in an emergency; what family rules; routines and expectations changes; how family holidays, birthdays, and other life events will be handled; how communication will be maintained during deployment; strategies that can be utilized to maintain a trusting relationship during separation.

Emotional chaos and making plans were reported by the wives in this study as difficult, and at times, overwhelming for the entire family. As their husbands were preparing for the deployment, both wives' and husbands' workload increased tremendously. While maintaining their civilian employment, both were fulfilling military training requirements, helping the family to prepare for separation, completing wills and powers-of-attorney, and tying up multiple lose ends [1].

The second phase occurred during deployment and yielded five themes. Themes of taking the reigns, placing focus elsewhere, emotional and physical turmoil, staying strong, and reaching out were found to be reflected in previous literature. During deployment, several women with young children related there was no time for themselves. The literature clearly reflects the wife being the sole responsible adult with little relief, and several authors have reported this in the past [5, 9, 18].

The next theme identified was placing focus elsewhere. The wives felt as if they needed to avoid television and radio accounts of military actions. It was as if watching or listening was simply too real and caused additional anxiety. The author found this especially evident when children were involved, and the wife would “protect them” by avoiding the news. Intentional energy was directed towards engaging in work or other activities to avoid media portrayals of war. Lapp et al. [19] found that military wives avoided the media as a form of self-care.

Emotional and physical turmoil was evident during the interviews. During the deployment phase, wives experience a wide range of emotions, including sadness, helplessness, crying, anxiety, depression, despair, guilt, low self-esteem, detachment, anger, intolerance for their children, fear of their husband's infidelity, and suicidal ideations [2, 20]. Wives and family members may also feel lonely, restless, and fearful for the safety of the deployed service member, along with having trouble sleeping [2, 10, 15, 20]. Other physical symptoms may also occur, such as fatigue, headaches, inability to cope, poor concentration, menstrual problems, weight changes, back pain, headaches, lethargy, and compromised adaptation [2, 21, 22]. A large concern for many of the wives was how the deployment would change their husband.

In addition, finances may be a significant source of stress, as military pay may not match civilian pay, and the wives may have difficulty finding affordable child care [16]. The additional financial resources that the military provides, such as deployment allowances and combat zone tax advantages, may not be enough to sustain a household, creating additional stress [16].

During deployment, several wives reported extreme loneliness. Personal contact from a volunteer, the family readiness team, or unit representatives was found to help reduce feelings of loneliness and help military wives and families cope better with deployment [16]. Education about the stress of separation, depression, coping, and adaptation may reduce the wife's feeling that she cannot cope [22]. Participation in support groups has been shown to assist with the coping skills needed during deployment [22, 23].

Reaching out was a theme that emerged when women discussed their coping with deployment. They reached out not only to their military spouse via email and phone calls, but also to the military resources, family, friends, and spiritual community. This communication helps sustain a healthy relationship and maintains the family's mental health [16, 19]. Family readiness groups provide information and assistance to help families cope and deal with the deployment successfully and offer websites to help the family feel connected [16]. Family and friends also act as a support system during deployment and many times assist with household maintenance and child care.

Returning home is a particularly important and stressful time for the wife. The longer the deployment lasts, the more wives worry about the effects it will have on family dynamics [16]. Family roles and power dynamics may have changed, and the returning veteran needs to reintegrate into the family unit [1, 15, 16, 24]. Problems can result when the husband expects things to be the same when he returns [3]. Reunion adjustment can bring on anxiety, self-doubt, insecurity, and resentment over the loss of independence [1]. In one study, three-fourths of survey respondents found that the first 3 months after their husband's return were the most stressful [16]. Communication can be temporarily strained when the returning soldier questions how or why things were done the way they were during deployment [15]. It can take time and patience to reestablish sexual intimacy. In fact, some military wives felt the need to be courted again [22]. The wife left at home may resent the returning service member, feeling that deployment was more exciting than being at home [21].

Many military personnel have difficulty separating from the combat mission and may turn to domestic violence [1]. Hardships that the family faces during this phase may include marital or family problems, financial trouble, potential infidelity, and mental or physical health issues [1].

## 6. Suggestions for Practice

The wives of deployed soldiers face many stressors, from the initial news of deployment, during deployment, and after deployment. They are often the silent partners trying to manage the home, family, and work environments. Before and during deployment, wives spoke of emotional chaos and turmoil. This was manifested as physical and/or emotional symptoms. The military recognizes these experiences occur and reports that family stress can be mild to debilitating and can affect their physical and emotional health [25]. Nurses, physicians, and other healthcare providers can give better care by understanding how the different phases of deployment and separation affect the wife's coping ability, her physical and emotional health, and her relationships. The healthcare practitioner's objective might help the wife acclimate to the change in her life and her role in the healthiest way possible [8, 18]. The healthcare professional may want to complete a thorough psychosocial history, including asking questions about any changes in family dynamics and finding out what support systems are available [9, 26]. The healthcare practitioner should assess the wife's stress level looking for signs of depression, anxiety, loneliness, sadness, resentment, anger, sleep disturbances, changes in eating patterns, and feelings of being overwhelmed with family and household responsibilities. It is important to reassure the wife that her feelings are normal, but also unique and distinct [9]. Recognizing and normalizing the stressful effects of deployment can provide reassurance and lessen anxiety. Providing education about the body's physical response to stress [9] and offering strategies for adaptation and coping are also beneficial [19, 24]. These wives are attempting to create normalcy for their family and for themselves. By understanding the phases of the deployment, the healthcare provider is able to assist them in knowing that it is normal to feel denial, anger, and sadness.

During deployment wives spoke of taking the reins and actively taking charge of many aspects of the family. These wives need to be encouraged to take an active role in promoting their own mental health and physical well-being. Listed are specific suggestions to assist wives in coping, nurturing themselves, and reducing their stress. Some suggestions come from the wives in the present study and others are from general health recommendations.

- Maintain their support network of family and friends.
- Turn the TV, radio, or Internet off before the news becomes overwhelming.
- Establish a healthy routine, such as eating nutritious meals.
- Obtain adequate sleep and practice relaxation.
- Exercise daily to lower adrenaline levels and promote healthy sleep.
- Keep calm by praying, listening to music, meditating, journaling, or doing yoga.
- Do at least one activity daily that brings joy or happiness.

To help reduce the risk of depression, the wife can be referred to support services, such as the Family Readiness Unit, support groups, and mental health services. In situations where depression and anxiety are moderate to severe, the use of antidepressants may be helpful. Emotional support should be offered before, during, and after the husband returns home. The healthcare practitioner is in a position to positively impact these wives by normalizing the stressful effects of deployment and making life-changing suggestions, which these wives can implement to reduce stress and improve their health and well-being.

## 7. Limitations

The results are not generalizable but reflect a body of unique stories reflecting the specific sample. The study was limited by the small number of White, well-educated, female participants living in the Midwest, and findings would be more generalizable if repeated with a different sample. Additionally, all participants were wives of Army Reserve soldiers, and the results might be different if soldiers from other military branches were represented or if the soldiers were career-bound instead of reservists.

## 8. Research Implications

Further research investigating larger numbers of diverse military family experiences would be interesting. Also, studying the experiences of reintegration of the husband into the family needs further work. In order to establish reliability, the researcher sought input from participants regarding the results. This is referred to as member checking. Three participants shared concerns about this phase in particular. All three felt they were inadequately prepared for reestablishing roles within the family. The stress experienced within the family was not anticipated and involved seemingly simply day-to-day activities, such as loading the dishwasher or paying bills, but also blossomed into much larger conflicts surrounding disciplining children, who may not have had a previous or at least recent relationship with the father returning from deployment. Women felt the healthcare system was not prepared for their needs, so research investigating the knowledge and preparation of healthcare practitioners is needed.

## Figures and Tables

**Table 1:** Phases of deployment.

Phase	Properties	Themes
News of deployment	Awareness	Emotional chaos
		Making preparations
Deployment	Engagement	Taking the reins
		Placing focus elsewhere
	Change and difference	Emotional and physical turmoil
		Staying strong
		Reaching out
After deployment	Time span	Absence makes the heart grow fonder
	Critical points and events	Reestablish roles
